# Paeonol Protects against Methotrexate Hepatotoxicity by Repressing Oxidative Stress, Inflammation, and Apoptosis—The Role of Drug Efflux Transporters

**DOI:** 10.3390/ph15101296

**Published:** 2022-10-21

**Authors:** Mohamed A. Morsy, Rania Abdel-Latif, Sara Mohamed Naguib Abdel Hafez, Mahmoud Kandeel, Seham A. Abdel-Gaber

**Affiliations:** 1 Department of Pharmaceutical Sciences, College of Clinical Pharmacy, King Faisal University, Al Hofuf 31982, Al-Ahsa, Saudi Arabia; 2 Department of Pharmacology, Faculty of Medicine, Minia University, El-Minia 61511, Egypt; 3 Department of Pharmacology & Toxicology, Faculty of Pharmacy, Minia University, El-Minia 61511, Egypt; 4 Department of Histology and Cell Biology, Faculty of Medicine, Minia University, El-Minia 61511, Egypt; 5 Department of Biomedical Sciences, College of Veterinary Medicine, King Faisal University, Al Hofuf 31982, Al-Ahsa, Saudi Arabia; 6 Department of Pharmacology, Faculty of Veterinary Medicine, Kafrelsheikh University, Kafr El-Sheikh 33516, Egypt

**Keywords:** methotrexate, hepatotoxicity, paeonol, oxidative stress, inflammation, P-gp, Mrp-2

## Abstract

Methotrexate (MTX) is an effective chemotherapeutic agent against a wide range of tumors and autoimmune diseases; however, hepatotoxicity limits its clinical use. Oxidative stress and inflammation have been implicated in the pathogenesis of MTX-induced hepatotoxicity. Paeonol is a natural phenolic compound reported for its antioxidant and anti-inflammatory properties. The current study aimed to investigate the protective effect of paeonol against MTX-induced hepatotoxicity in rats and various mechanisms that underlie this postulated effect. Paeonol was administered orally in a dose of 100 mg/kg, alone or along with MTX, for 10 days. Hepatotoxicity was induced via a single intraperitoneal dose of MTX (20 mg/kg) on day 5 of the experiment. Concomitant administration of paeonol with MTX significantly ameliorated distorted hepatic function and histological structure, restored hepatic oxidative stress parameters (MDA, NO, and SOD), and combated inflammatory response (iNOS and TNF-α). Additionally, paeonol enhanced cell proliferation and survival, evidenced by upregulating the proliferating cell nuclear antigen (PCNA) and suppressing apoptosis and the disposition of collagen fibers in rat livers treated with MTX. Importantly, paeonol upregulated the drug efflux transporters, namely P-glycoprotein (P-gp) and the multidrug resistance-associated protein 2 (Mrp-2) in MTX-treated rats. In conclusion, paeonol offered a potent protective effect against MTX-induced hepatotoxicity through suppressing oxidative stress, inflammation, fibrosis, and apoptosis pathways, along with P-gp and Mrp-2 upregulation.

## 1. Introduction

Methotrexate (MTX) is a widely used chemotherapeutic agent for treating various malignant tumors and autoimmune diseases [1,2]. Unfortunately, MTX use is usually accompanied by multi-organ damage, of which hepatotoxicity is the most prominent [3]. Liver injury progresses into fibrosis, and cirrhosis is reported in up to 50% of patients with relatively high doses of MTX [4,5]. Although the mechanism of MTX-induced hepatotoxicity is not yet fully understood, MTX induces hepatotoxicity by triggering oxidative, inflammatory, fibrotic, and apoptotic signaling [6]. MTX-induced intracellular reactive oxygen species trigger pro-inflammatory signals such as the expression of the inducible nitric oxide synthase (iNOS) and the release of tumor necrosis factor-α (TNF-α) [7,8].

MTX-elicited persistent inflammation usually evokes hepatic fibrosis via the synthesis of the extracellular matrix and hepatic fibrogenesis [9,10]. Furthermore, prolonged intracellular accumulation of MTX predisposes hepatic cell death and fibrosis via depleting hepatic folate levels, interfering with DNA synthesis in hepatocytes, and stimulating collagen synthesis [3,5]. Importantly, many protein transporters govern the MTX hepatic uptake and subsequently modulate MTX hepatic accumulation and toxicity [11]. Cellular protection against xenobiotics involves the efflux of such harmful molecules via transmembrane transporters, including P-glycoprotein (P-gp), and the multidrug resistance-associated protein 2 (Mrp-2), which facilitate their excretion and suppress their cellular uptake [12]. Therefore, modulations of these transporters could be a potential target for an effective cytoprotective mechanism against cellular injury mediated by MTX hepatic accumulation.

Paeonol is a natural phenolic compound that has been shown to have significant antioxidant and anti-inflammatory properties [13,14], along with reported potent protective effects in various disease models [13,15,16]. Paeonol, among other various natural products, has displayed multiple drug resistance (MDR) regulations via a mechanism related to the modulation of drug efflux transporters [17,18]. Various reports have recommended the use of MDR-targeted natural products as an adjuvant therapy with chemotherapeutic agents to reduce chemotherapy-induced toxicities. However, no previous study has evaluated the role of paeonol as an MTX-adjuvant therapy in reducing the potential hepatotoxicity effect. Therefore, this study was designed to investigate the hepatoprotective effects of paeonol in the MTX-induced hepatotoxicity model, and to uncover the mechanisms underlying such protection, focusing on the role of drug efflux transporters, namely P-gp and Mrp-2 in this postulated protective effect.

## 2. Results

### 2.1. Effect on Liver Function and Hepatic Oxidative Status

**Figure 1:**
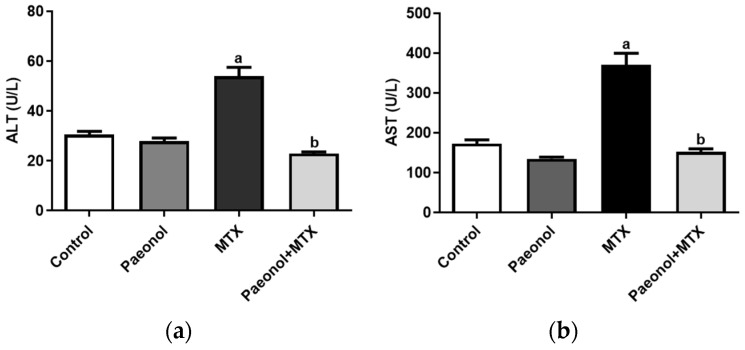
The effect of paeonol on the serum (**a**) alanine transaminase (ALT) and (**b**) aspartate transaminase (AST) in methotrexate (MTX)-induced hepatotoxicity in rats. Data are represented as mean ± SEM (*n* = 6). Letters a,b Denote significant differences from the normal control and the MTX groups, respectively, at *p* ˂ 0.05.

**Figure 2:**
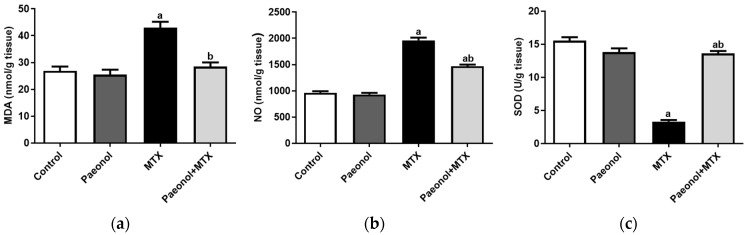
The effect of paeonol on hepatic (**a**) lipid peroxide malondialdehyde (MDA), (**b**) nitric oxide (NO), and (**c**) superoxide dismutase (SOD) in MTX-induced hepatotoxicity in rats. Data represent the mean ± SEM of 6 observations. Letters a,b Denote significant differences from the normal control and the MTX groups, respectively, at *p* ˂ 0.05.

The administration of MTX significantly increased serum levels of alanine transaminase (ALT) and aspartate transaminase (AST) compared with the control group, in which it was prevented in paeonol-pretreated rats (Figure 1). Treating the rats with paeonol alone showed that the values of the hepatic functions and oxidative stress parameters are comparable to those of the control group. However, hepatic malondialdehyde (MDA) and NO levels were significantly increased alongside a reduced superoxide dismutase (SOD) activity in the MTX group compared with the control group, which was completely mitigated by paeonol treatment before the MTX challenge (Figure 2).

### 2.2. Effect on Histopathology Examination

**Figure 3:**
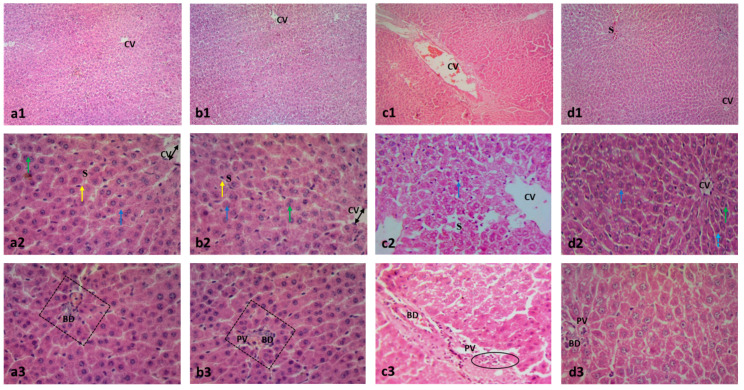
Photomicrographs showing the effect of paeonol on rat liver tissues stained with H&E in methotrexate (MTX)-induced hepatotoxicity (×100 and ×400). The tissue of the control group (**a1**) showed typical morphological features of hepatic cells with normal lobular architecture and normal central veins (CV). Similarly, the liver tissues of the paeonol group (**b1**) showed a normal hepatic structure. The liver tissues of both the control and paeonol groups (**a2**,**b2**) showed central veins (CV) which appeared to be lined by flat endothelial cells (double arrows), surrounded by cords of polygonal hepatocytes with granular cytoplasm and central, rounded, vesicular nuclei (blue arrows). Some cells appeared to be binucleated (green arrows). The blood sinusoids (S) are lined by Kupffer cells (yellow arrows). Additionally, the investigated liver tissues of the two groups showed branches of portal veins (PV) and bile ductules (BD) seen at a portal area (squares) (**a3**,**b3**). The liver tissues of the MTX-treated rats showed a disturbed lobular architecture with dilated central veins (CV) and blood sinusoids (S), and the cells have darkly stained nuclei with deeply stained cytoplasm (blue arrow) (**c1**,**c2**). Moreover, the liver tissues of the MTX-treated rats showed dilated bile ductules (BD) and a dilated portal vein (PV) surrounded by cellular infiltrations (circle) (**c3**). The microscopic examination of the liver tissues of the paeonol + MTX group showed a normal lobular architecture with apparently normal central veins (CV), except for a focal congested blood sinusoid (S) (**d1**). In the same group, the hepatocytes appeared to be polygonal with acidophilic cytoplasm and vesicular nuclei (blue arrow) (**d2**). Furthermore, the liver tissues of the paeonol + MTX group showed some binucleated cells (green arrow) and a few darkly stained nuclei with deeply stained cytoplasm (**d2**). In the same group, the portal vein (PV) and bile ductules (BD) have a normal appearance (**d3**).

**Figure 4:**
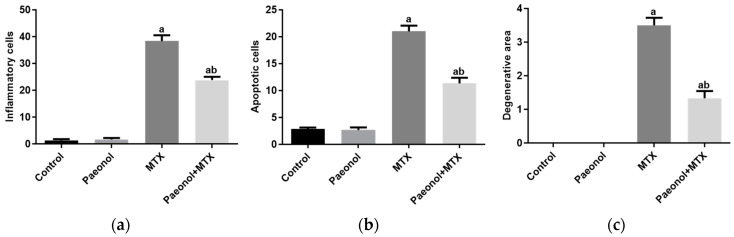
The effect of paeonol on the severity of histopathological lesions, (**a**) inflammatory cells, (**b**) apoptotic cells, and (**c**) degenerative area, in methotrexate (MTX)-induced hepatotoxicity in rats. Data are represented as mean ± SEM (*n* = 6). Letters a,b Denote significant differences from the normal control and the MTX groups, respectively, at *p* ˂ 0.05.

The MTX significantly distorted the liver architecture and increased the numbers of inflammatory and apoptotic cells and the degenerative area of hepatic cells compared to the control group. The paeonol pretreatment preserved the normal liver structure with a significant decrease in cellular inflammation, apoptosis, and degeneration in the MTX-challenged rats. It is worth noting that the hepatic structure of the liver tissues of the rat group treated only with paeonol was similar to that of the control group (Figure 3 and Figure 4).

**Figure 5:**
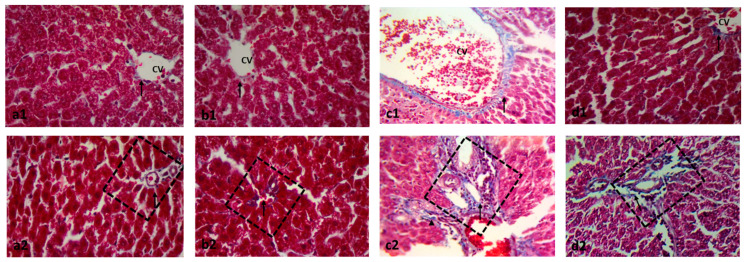

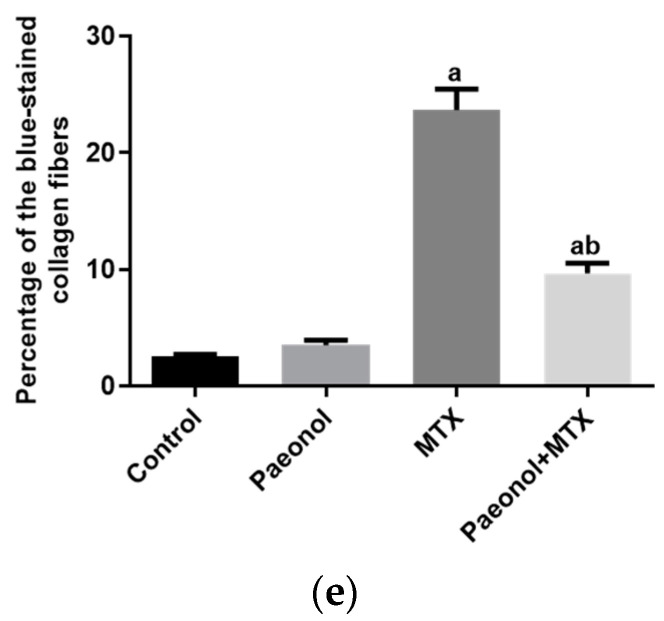
Photomicrographs showing the effect of paeonol on rat liver tissues stained with Masson’s trichrome in methotrexate (MTX)-induced hepatotoxicity (×400). The liver tissues of the control (**a1**,**a2**) and paeonol (**b1**,**b2**) groups show a minimal amount of blue-stained collagen (arrows), especially around the central veins (CV) and portal area (squares). On the contrary, the liver tissues of the MTX-treated rats show an apparent increase in blue-stained collagen deposits (arrows) around the central veins (CV) and the portal area (square), with the appearance of collagen fibers running between and surrounding the hepatocytes (arrowhead) (**c1**,**c2**). In contrast, the liver tissues of the paeonol + MTX group show a minimal amount of collagen fibers (arrows) around the central veins (CV) and portal area (square) (**d1**,**d2**). A semi-quantitative analysis of the collagen fibers (**e**) summarizes the results of six experiments as mean ± SEM. Letters a,b Denote significant differences from the normal control and the MTX groups, respectively, at *p* ˂ 0.05.

Masson’s trichrome stain in Figure 5 demonstrates the normal distribution of collagen fibers around central veins, and fine threads of collagen fibers were detected around the hepatic portal vein of control and paeonol groups. However, the untreated MTX group showed increased collagen fiber deposition between the hepatocytes and pericentral and periportal zones. The liver tissues of the paeonol + MTX group displayed a significant regression in the collagen fiber deposition in such areas (Figure 5).

### 2.3. Effect on Immunohistochemistry and ELISA

**Figure 6:**
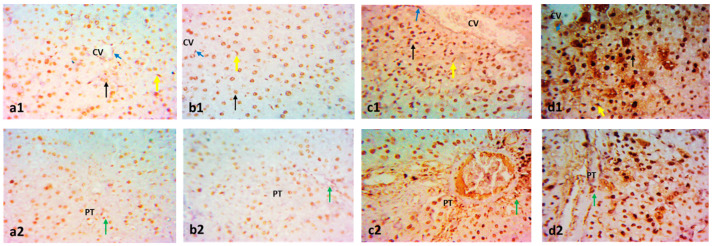

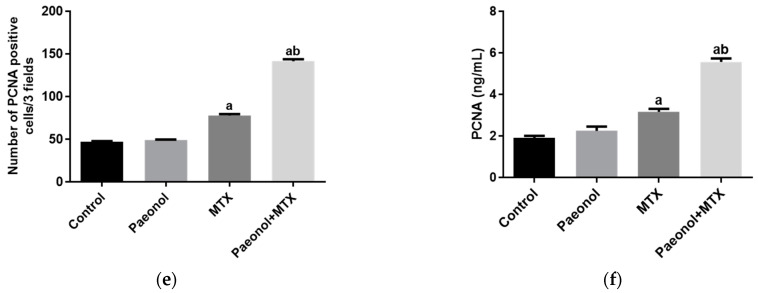
The immunohistochemical examination of hepatic proliferating cell nuclear antigen (PCNA) expression in methotrexate (MTX)-induced hepatotoxicity (×400). The liver tissues of the control (**a1**,**a2**) and paeonol (**b1**,**b2**) groups show hepatocytes (black arrows) and Kupffer cells (yellow arrows) with PCNA-positive nuclei. Notice the positive endothelial cells lining the central veins (CV) (blue arrows) and portal tracts (PT) (green arrows). The liver tissues of the MTX-treated rats show apparent numerous PCNA-positive nuclei in hepatocytes (black arrow), Kupffer cells (yellow arrow), and endothelial cells lining the CV (blue arrow) and the PT (green arrow) with intense nuclear expression (**c1**,**c2**). The liver tissues of the paeonol + MTX group show more numerous PCNA-positive nuclei in the same cells (**d1**,**d2**). A semi-quantitative analysis of PCNA positive cells (**e**). Hepatic PCNA level using PCNA ELISA kit (**f**). Data are represented as mean ± SEM (*n* = 6). Letters a,b Denote significant differences from the normal control and the MTX groups, respectively, at *p* ˂ 0.05.

**Figure 7:**
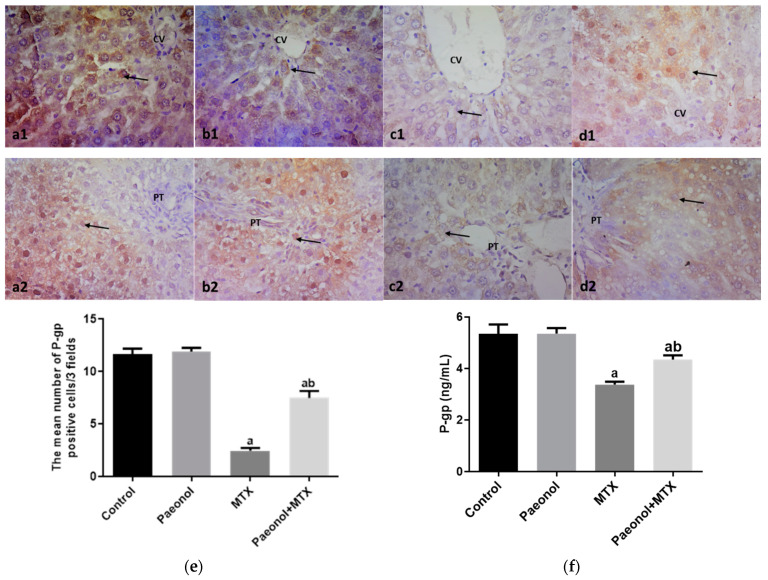
The immunohistochemical examination of hepatic P-glycoprotein (P-gp) expression in methotrexate (MTX)-induced hepatotoxicity (×400). The liver tissues of the control (**a1**,**a2**) and paeonol (**b1**,**b2**) groups show nuclear P-gp expression (arrows) in hepatocytes surrounding the central veins (CV) and portal areas (PT). The liver tissues of the MTX-treated rats show few nuclear P-gp expressions (arrows) (**c1**,**c2**). On the contrary, the liver tissues of the paeonol + MTX group show many nuclear P-gp expressions (arrows) in hepatocytes surrounding the CV and PT (**d1**,**d2**). A semi-quantitative analysis of P-gp positive cells (**e**). Hepatic P-gp level using P-gp ELISA kit (**f**). Data are represented as mean ± SEM (*n* = 6). Letters a,b Denote significant differences from the normal control and the MTX groups, respectively, at *p* ˂ 0.05.

**Figure 8:**
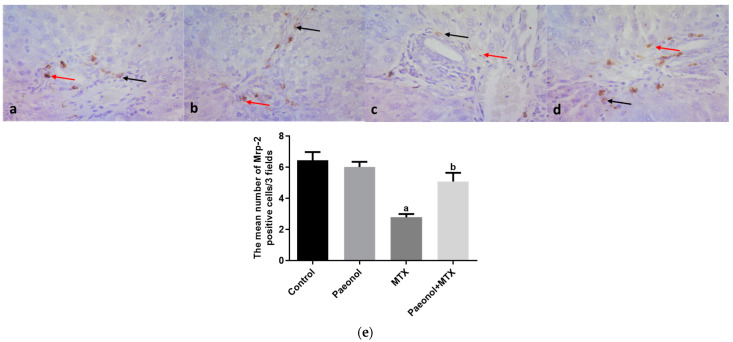
The immunohistochemical examination of hepatic multidrug resistance-associated protein 2 (Mrp-2) expression in methotrexate (MTX)-induced hepatotoxicity (×400). The liver tissues of the control (**a**) and paeonol (**b**) groups show cytoplasmic (black arrows) and nuclear (red arrows) Mrp-2 expression in the portal tract cells, mainly in the cells of bile ductules. The liver tissues of the MTX-treated rats show little cytoplasmic (black arrow) and nuclear (red arrow) Mrp-2 expression in the previously mentioned cells (**c**). In contrast, the liver tissues of the paeonol + MTX group show marked cytoplasmic (black arrow) and nuclear (red arrow) Mrp-2 expression in the portal tract cells, mainly in the cells of bile ductules (**d**). A semi-quantitative analysis of Mrp-2 positive cells (**e**). Data are represented as mean ± SEM (*n* = 6). Letters a,b Denote significant differences from the normal control and the MTX groups, respectively, at *p* ˂ 0.05.

**Figure 9:**
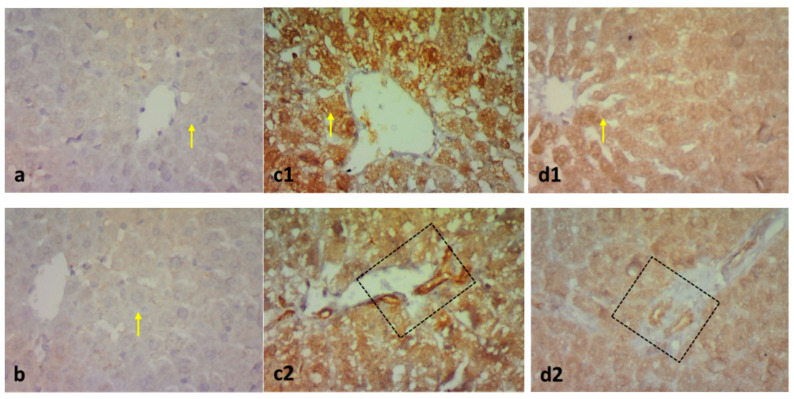

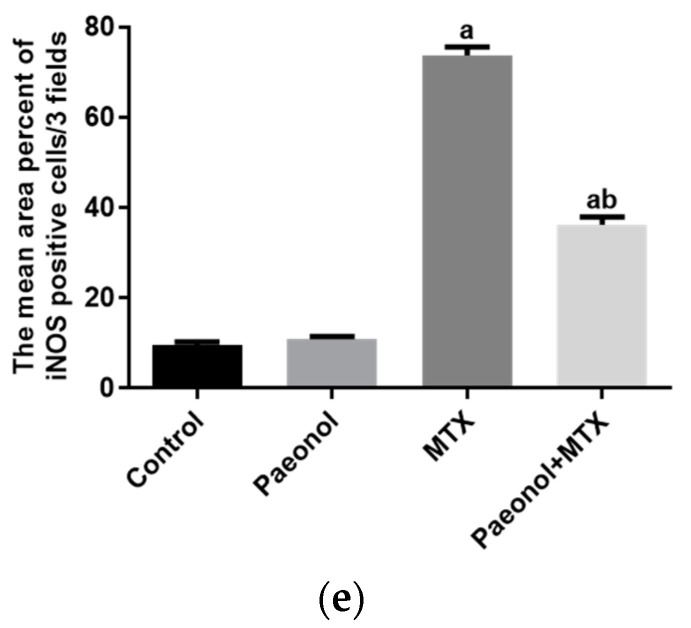
The immunohistochemical examination of hepatic inducible nitric oxide synthase (iNOS) expression in methotrexate (MTX)-induced hepatotoxicity (×400). The liver tissues of the control (**a**) and paeonol (**b**) groups show hepatocytes with faint cytoplasmic iNOS expression (yellow arrows). The liver tissues of the MTX-treated rats show hepatocytes with intense cytoplasmic iNOS expression (yellow arrow) (**c1**). Expression is also noticed in the portal area (square) (**c2**). In contrast, the liver tissues of the paeonol + MTX group show hepatocytes with little iNOS expression (yellow arrow) (**d1**). Little expression is also noticed in the portal area (square) (**d2**). A semi-quantitative analysis of iNOS positive cells (**e**). Data are represented as mean ± SEM (*n* = 6). Letters a,b Denote significant differences from the normal control and the MTX groups, respectively, at *p* ˂ 0.05.

**Figure 10:**
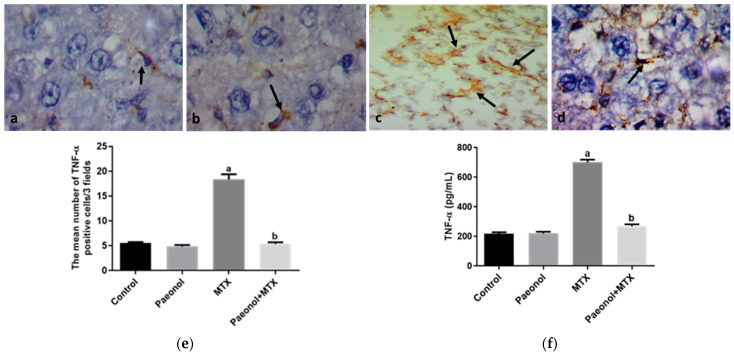
The immunohistochemical examination of hepatic tumor necrosis factor-α (TNF-α) expression in methotrexate (MTX)-induced hepatotoxicity (×1000). The liver tissues of the control (**a**) and paeonol (**b**) groups show Kupffer cells with TNF-α cytoplasmic expression (arrows). The liver tissues of the MTX-treated rats show many Kupffer cells with TNF-α cytoplasmic expression (arrows) (**c**). In contrast, the liver tissues of the paeonol + MTX group show few Kupffer cells with TNF-α cytoplasmic expression (arrow) (**d**). A semi-quantitative analysis of TNF-α positive cells (**e**). Hepatic TNF-α level using TNF-α ELISA kit (**f**). Data are represented as mean ± SEM (*n* = 6). Letters a,b Denote significant differences from the normal control and the MTX groups, respectively, at *p* ˂ 0.05.

The hepatic protein expression of proliferating cell nuclear antigen (PCNA) was significantly increased (*p* < 0.05) in rats that received MTX in comparison with the control group (Figure 6). On the contrary, the hepatic expressions of P-gp and Mrp-2 measured in the MTX-challenged group showed significant downregulations (*p* < 0.05) compared with the control group. The hepatic expression of the previous markers was comparable in both control and paeonol groups. However, treatment with paeonol significantly increased (*p* < 0.05) PCNA, P-gp, and Mrp-2 expressions in the MTX-challenged rats compared to the non-treated MTX rats (Figure 6, Figure 7 and Figure 8). Additionally, we assessed the protein expression levels of iNOS and TNF-α (Figure 9 and Figure 10). The normal and control paeonol-treated hepatic tissues revealed negative iNOS and TNF-α immunohistochemical staining. The MTX-intoxicated group showed a significantly higher cytoplasmic expression for both parameters in the rat hepatocytes than in the control group, which was lowered considerably by paeonol treatment. Regarding hepatic PCNA, P-gp, and TNF-α, the same results were obtained using PCNA, P-gp, and TNF-α ELISA kits, respectively (Figure 6f, Figure 7f and Figure 10f).

### 2.4. Effect on Hepatic Apoptosis

**Figure 11:**
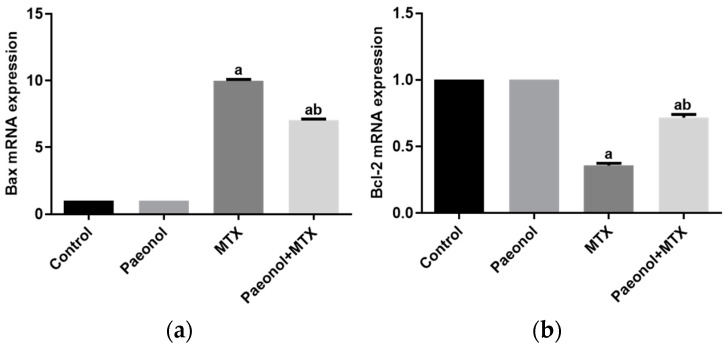
The effect of paeonol on hepatic mRNA expressions of Bax (**a**) and Bcl-2 (**b**) in methotrexate (MTX)-induced hepatotoxicity in rats. Data are represented as mean ± SEM (*n* = 6). Letters a,b Denote significant differences from the normal control and the MTX groups, respectively, at *p* ˂ 0.05.

The real-time polymerase chain reaction (PCR) experiments indicated that MTX significantly increased hepatic Bcl-2-associated X protein (Bax) mRNA. At the same time, it decreased the expression of B-cell lymphoma 2 (Bcl-2) compared with the control group. Treating the rats with paeonol before MTX administration significantly reduced the upregulated Bax mRNA expression and increased Bcl-2 mRNA expression. Treatment with paeonol alone showed a similar effect in the control group for Bax and Bcl-2 mRNA expressions (Figure 11).

## 3. Discussion

Hepatotoxicity is a significant adverse effect of MTX, limiting its clinical use. Thus, approaches for using potential adjuvant therapies with hepatoprotective activities are widely adopted during cancer chemotherapy [17]. We demonstrated that paeonol, an antioxidant flavonoid derivative, protected against MTX-induced hepatotoxicity. In line with previous reports [13,19], the current results indicate that challenging the rats with MTX instigated hepatic histopathological damage and elevated the serum levels of liver function enzymes. However, pretreatment with paeonol prevented the MTX-induced liver damage, as shown by its ameliorating effect on serum liver function enzymes and the prevention of all histological alterations. Accumulating evidence suggests that the MTX-induced cytotoxicity and tissue damage involves oxidative stress and the activation of reactive oxygen species-mediated signaling [20,21]. Our results support the studies showing an imbalance in the oxidative status in vivo in response to MTX treatment. We reported a significant suppression in hepatic SOD activities alongside increased MDA levels, a marker of lipid peroxidation, and NO levels in the liver tissues following an MTX challenge. When NO reacts with superoxide radicals, they generate the deleterious peroxynitrite that damages DNA; thus, it contributes to the induction of apoptosis [22]. Studies have shown that MTX induces apoptosis via activating the mitochondrial intrinsic apoptotic pathway [23]. This mechanism is confirmed by the results of the current study showing upregulation of the pro-apoptotic gene expression Bax in the liver tissues of MTX-treated rats. The present findings depict that the MTX-induced apoptotic changes involved the activation of the intrinsic pathway by regulating the Bcl-2 family of proteins, consistent with the observed upregulation of Bax gene expression.

Fascinatingly, the pretreatment of rats with daily paeonol attenuated the MTX-induced liver toxicity, with the potent mitigation of hepatic oxidative stress induced by the MTX treatment, as evident by the suppression of lipid peroxidation and the NO hepatic content along with the resorting of the SOD activity. Several previous studies have reported similar antioxidant effects of paeonol in different tissues, such as the kidney, heart, stomach, and testis [13,14,16,24,25]. Given its efficacy in diminishing oxidative stress, paeonol prevents the hepatocyte death triggered by MTX. The results of the current study show that paeonol pretreatment suppressed the pro-apoptosis Bax and upregulated Bcl-2, which has been attributed to its direct antioxidative properties [13,14].

PCNA is essential in cell cycle regulation and DNA repair and replication [26]. PCNA expression has been used as a potential marker for the proliferative activity of the tissues, and it could be used as a biomarker for the diagnosis and prognosis of different malignant tumors [27,28]. In the current study, PCNA expression levels in the liver tissues of MTX-treated rats showed a significant increase compared to controls. These results may be attributed to the stimulation of hepatocyte proliferation as a compensatory reaction in response to the tissue damage caused by MTX administration [29]. Direct inhibition of DNA replication is usually associated with MTX therapy, and this may explain the MTX-induced depletion in PCNA expression observed by other studies [30,31,32]. The variability in MTX treatment regimens could be a reasonable cause for these contradictory observations of the MTX effect on PCNA expression. Alternatively, the current results show that paeonol pretreatment in MTX-challenged rats revealed strongly positive PCNA staining reactivity of the hepatocyte nuclei, indicating improved cell renewal. The upregulation of PCNA contributes to cell proliferation and survival via promoting cell cycle progression and the reduction of apoptosis [33]. Previous studies have associated the protective effect of paeonol against the harmful effects of MTX with suppression of the reactive oxygen species, DNA fragmentation, and apoptosis [13,24]; thereby, explaining the ability of paeonol to reverse tissue damage and initiate cell proliferation to promote the healing of damaged tissues.

MTX, like other chemotherapeutic agents, may provoke systemic inflammation via the induction of intracellular reactive oxygen species, which activates a pro-inflammatory response involving iNOS and TNF-α upregulation [7,8], which is in line with our current findings. Furthermore, the present study shows an increase in collagen deposition in the hepatic tissue of MTX-treated rats, which predominantly induces hepatic fibrosis. The MTX-induced hepatic inflammatory response has been linked to hepatic stellate cell activation and extracellular matrix synthesis, which further accelerates fibrosis [34]. Attenuating hepatic inflammatory markers by paeonol treatment could explain the downregulation of collagen disposition reported in the liver tissues of the paeonol + MTX rat group. Previous studies [13,14] have shown that the anti-inflammatory properties of paeonol involved the modulation of iNOS and TNF-α, which is in accord with the present results. Of note, many studies have demonstrated that the upregulation of TNF-α is related to cell apoptosis via the regulation of the expression of the proapoptotic and antiapoptotic factors of the Bcl-2 protein family [35,36]. Therefore, in the current study, the inhibition of hepatic TNF-α levels that was associated with the paeonol treatment might trigger the antiapoptotic effect of paeonol.

P-gp and Mrp-2 are efflux transporters that decrease the intracellular accumulation of a drug or a toxicant in several organs, including the liver [12,37]. The current results show that MTX significantly reduced the hepatic expression of P-gp and Mrp-2, which agrees with previous studies [37,38]. Therefore, the paeonol-induced increase in the expression of P-gp and Mrp-2 proteins provides a plausible explanation for its hepatoprotective effects against MTX-induced injury by increasing its efflux. In agreement with the current results, our previous study [25] emphasized the upregulation of P-gp in the testis as a protective effect of paeonol against MTX-induced injury. Therefore, it could be concluded that paeonol-induced upregulation of efflux transporters in the liver offers a beneficial effect against MTX hepatotoxicity.

## 4. Materials and Methods

### 4.1. Chemicals

The paeonol was obtained from Sigma-Aldrich (St. Louis, MO, USA), and the MTX was obtained from Minapharm Pharmaceuticals (Cairo, Egypt). Kits for determining serum ALT and AST were purchased from Biodiagnostic (Giza, Egypt). In addition, the PCNA antibody was procured from Novus Biologicals (Centennial, CO, USA), the iNOS antibody was from Thermo Fisher Scientific (Waltham, MA, USA), the TNF-α antibody was from ABclonal (Woburn, MA, USA), while the P-gp and Mrp-2 antibodies were from Santa Cruz Biotechnology (Dallas, TX, USA). The other chemicals were purchased from local commercial sources and were of analytical grade.

### 4.2. Experimental Design

Twenty-four male Wistar rats (180–210 g) were purchased from the National Research Center (Giza, Egypt) and kept under standard laboratory conditions (23–25 °C, 55 ± 5% relative humidity, and 12 h light/dark cycle). The rats had free access to standard laboratory animal chow and tap water. After acclimatization for one week, the animals were randomly divided into four groups (*n* = 6). The first group (control) received only the vehicle. The second group (Paeonol) received a daily dose of paeonol (100 mg/kg, orally), freshly prepared in a 1% aqueous solution of carboxymethyl cellulose, for ten days [13,39]. The third group comprised the MTX-induced hepatotoxicity group (MTX), in which the rats received a single i.p. injection of MTX (20 mg/kg) on day 5 of the experiment [40]. Finally, the rats in the fourth group (Paeonol + MTX) received oral daily paeonol (100 mg/kg) for ten days and a single i.p. dose of MTX (20 mg/kg) on the fifth day of the experiment (Table 1).

### 4.3. Sample Preparation

At the end of the experiment, rats were euthanized to collect blood and liver samples. Serum was collected and stored at −80 °C after blood clotting and centrifugation at 5000 rpm for 15 min. Pieces of the liver were rapidly excised, rinsed in cold saline, then perfused thoroughly with cold saline, fixed in 10% formalin, and processed for histology and immunohistochemistry. The rest of the liver was immediately frozen in liquid nitrogen and kept at −80 °C for further analysis. Tissue homogenates were prepared (10% *w*/*v*) in ice-cold phosphate buffer (0.01 M, pH 7.4), centrifuged (3000 rpm, 20 min), and the supernatant was collected for further assays.

### 4.4. Biochemical Analysis

#### 4.4.1. Determination of Liver Function Tests

Serum activities of ALT and AST were measured via commercially available kits according to the provided instructions.

#### 4.4.2. Determination of Hepatic Oxidative Stress Biomarkers

Hepatic tissue levels of MDA (nmol/g) were measured as a marker of oxidative stress. MDA, a major degradation product of lipid peroxides, reacts with thiobarbituric acid producing a pink chromogen that is measured spectrophotometrically at 535 nm [41]. As an indicator of nitrosative stress, NO was measured following the Griess reaction after nitrate reduction to nitrite and expressed in nmol/g. The intensity of the developed color was measured at 540 nm in a spectrophotometer [42]. Finally, the activity of hepatic SOD (U/g tissue) was determined colorimetrically at 420 nm based on its ability to inhibit the autoxidation of pyrogallol [43].

### 4.5. Histological Evaluation

The hepatic tissues were dehydrated after fixation overnight in formalin using an ascending alcohol gradient. Tissues were cleared in xylene, rapidly embedded in paraffin, and 5-µm-thick sections were processed for hematoxylin–eosin or Masson trichrome staining. The stained sections were examined by light microscopy (Olympus CX23LEDRFS1, Olympus, Tokyo, Japan) to detect histopathological changes and collagen deposition.

### 4.6. Immunohistochemical Examination

After deparaffinization and rehydration, the endogenous tissue peroxidase was blocked by hydrogen peroxide (3%, 5 min). Sections were incubated overnight with the primary antibodies against PCNA, iNOS, TNFα, P-gp, and Mrp-2. After washing in PBS for 5 min, biotinylated secondary antibodies were added for 30 min and washed with PBS for 3–5 min. Then, streptavidin-conjugated peroxidase was added for 35 min. The action of peroxidase on 3,3-diaminobenzidine (added for 5 min) produced a brown-colored product, which is proportional to the amount of original protein in the sample. Sections were countered-stained with hematoxylin [44].

### 4.7. ELISA Analysis

The hepatic levels of PCNA, P-gp, and TNFα were measured using the PCNA (FineTest, Wuhan, Hubei, China), P-gp (US Biological, Salem, MA, USA), and TNF-α (Sigma-Aldrich) ELISA kits according to the provided instructions.

### 4.8. Morphometric Analysis

For all stained sections, three non-overlapping fields of six different rats in each group were analyzed using the image analyzer Leica QwinV.3 software (Leica Microsystems, Wetzlar, Germany) for calculating: (a) the mean area percent of the blue-stained collagen fibers in Masson’s trichrome stained sections, and the iNOS immunostained cells and (b) the mean number of PCNA, P-gp, Mrp-2, and TNF-α immunostained cells, and inflammatory cells (neutrophils, lymphocytes, and eosinophils), apoptotic cells (cells with deeply stained cytoplasm and small dense eccentric nuclei), and degenerated areas (containing vacuolated cells) in liver tissues as mentioned previously [45].

### 4.9. Real-Time PCR Determination of Hepatic Bax and Bcl-2

Extraction of the total RNA from the liver tissues was carried out using the RiboZol Reagent (AMRESCO, Solon, OH, USA) following the given instructions. UV spectrophotometry at 260 nm determined the total RNA quality and yield. The SensiFAST^TM^ SYBR^®^ Hi-ROX One-Step Kit (Meridian Bioscience, Memphis, TN, USA) was used to prepare the real-time PCR reaction mixture (25 μL) containing 50 ng of template RNA from each sample and 70 nM of specific primers. The reaction was monitored in the Applied Biosystems 7500 Fast Real-Time PCR System (Foster City, CA, USA). The forward and reverse primers were: 5′-AGAGGCAGCGGCAGTGAT-3′ and 3′-GTATGAGTGCCATCCAGAGCAG-5′ for Bax [46], 5′-CTTTGTGGAACTGTACGGCCCCAGCATGCG-3′ and 5′-ACAGCCTGCAGCTTTGTTTCATGGTACATC-3′ for Bcl-2 [47], and 5′-GTCGGTGTGAACGGATTTG-3′ and 3′-CTTGCCGTGGGTAGAGTCAT-5′ for glyceraldehyde-3-phosphate dehydrogenase (GAPDH) [13]. Data were analyzed relative to GAPDH to calculate the relative gene expression of Bax and Bcl-2 [48].

### 4.10. Statistical Analysis

The experimental results (mean ± SEM) were analyzed by GraphPad Prism software (Version 8.1) (GraphPad Software Inc., San Diego, CA, USA) using a one-way analysis of variance (ANOVA) followed by Tukey–Kramer for comparing the means of different groups. The differences between any two means were significant if *p* < 0.05.

## Figures and Tables

**Table 1:** Timeline schedule of the treatment regimen. MTX: methotrexate.

Group	Treatment Regimen									
	Day 1	Day 2	Day 3	Day 4	Day 5	Day 6	Day 7	Day 8	Day 9	Day 10
**Control**	Vehicle 1 mL, p.o.	Vehicle 1 mL, p.o.	Vehicle 1 mL, p.o.	Vehicle 1 mL, p.o.	Vehicle 1 mL, p.o.	Vehicle 1 mL, p.o.	Vehicle 1 mL, p.o.	Vehicle 1 mL, p.o.	Vehicle 1 mL, p.o.	Vehicle 1 mL, p.o.
**Paeonol**	100 mg/kg, p.o.	100 mg/kg, p.o.	100 mg/kg, p.o.	100 mg/kg, p.o.	100 mg/kg, p.o.	100 mg/kg, p.o.	100 mg/kg, p.o.	100 mg/kg, p.o.	100 mg/kg, p.o.	100 mg/kg, p.o.
**MTX**					20 mg/kg, i.p.					
**Paeonol + MTX**	100 mg/kg, p.o.	100 mg/kg, p.o.	100 mg/kg, p.o.	100 mg/kg, p.o.	100 mg/kg, p.o. + 20 mg/kg, i.p.	100 mg/kg, p.o.	100 mg/kg, p.o.	100 mg/kg, p.o.	100 mg/kg, p.o.	100 mg/kg, p.o.

## Data Availability

Data is contained within the article.
